# Dynamical Buildup of Lasing in Mesoscale Devices

**DOI:** 10.1038/srep15858

**Published:** 2015-10-29

**Authors:** T. Wang, G. P. Puccioni, G. L. Lippi

**Affiliations:** 1 Institut Non Linéaire de Nice, Université de Nice-Sophia Antipolis; 2 UMR 7335 CNRS, 1361 Route des Lucioles, F-06560 Valbonne, France; 3 Istituto dei Sistemi Complessi, CNR, Via Madonna del Piano 10, I-50019 Sesto Fiorentino, Italy

## Abstract

The classical description of laser field buildup, based on time-averaged photon statistics of Class A lasers, rests on a statistical mixture of coherent and incoherent photons. Here, applying multiple analysis techniques to temporal streams of data acquired in the threshold region of a Class B mesoscale laser, we conclusively show that new physics is involved in the transition: the lasing buildup is controlled by large dynamical spikes, whose number increases as the pump is raised, evolving into an average coherent field, modulated by population dynamics, and eventually relaxing to a steady state for sufficiently large photon numbers. These results explain inconsistencies observed in small scale devices. Implications for nanolaser coherence properties, threshold identification and regimes of operation, including new potential applications, are discussed.

Coherence is the main distinctive feature which identifies laser emission. It has been thoroughly characterized in Class A lasers^1^ and used to assess the quality of the emitted radiation. Semiconductor-based (i.e., Class B) devices, due to their intrinsic time scales and the strong coupling of the emitters with their environment, possess a much more complex dynamics. This can be recognized in the photon-statistics-based measurements of the coherence for nanolasers, where an interpretation based on the traditional framework presents internal inconsistencies^2^,^3^,^4^,^5^,^6^,^7^. The problems currently open can be summarized as follows: 1) a clear picture of the evolution of the e.m. field coherence in the transition from spontaneous emission to lasing is missing for Class B lasers; 2) a meaningful definition of threshold in nanolasers^8^,^9^ and many new microcavity lasers^10^ is still unavailable.

A way out of this impasse requires the consideration of the full dynamical evolution of small-sized lasers, which couple the extreme volume reduction of nanocavities to the physics of the “phase transition” (threshold). In other words, we need to establish a connection between the quantum and statistical properties of light and the dynamical properties of small systems^8^. In this paper we show that the transition in Class B lasers is dominated by the *dynamics*, and that two interpretations of “coherence” hide behind the correlations. This contrasts the traditional Class A laser picture, where the lasing transition is described by a *time-independent, statistical mixture* of coherent and incoherent photons.

An overview of the current state of knowledge on the coherence buildup in lasers (Fig. 1) helps identifying the various facets of the problem. In order to present an overall picture we group the lasers by size and physical parameter values^11^ (electric field intensity for Class A, e.g., gas and dye lasers; field intensity and population for Class B, e.g., solid state and semiconductor lasers; field intensity, population and medium’s polarization for Class C, e.g., Far-InfraRed lasers). As detailed in the figure caption, a full, well-established characterisation of the transition to coherence exists only for Class A lasers, and its theoretical description has been widely used – for lack of viable alternatives – to interpret experimental results obtained in other laser classes. It is obvious, however, that further progress requires explicit consideration of those systems for which no well-established description exists.

Nanolasers are the most attractive devices to expand this description (Fig. 1). However, to this day the technological demands they impose on the measurement chain render a detailed investigation impossible (very low emitted photon numbers and very fast response time). Thus, sensible studies can only be carried out by turning to the mesoscale, quantitatively characterized by the fraction of spontaneous emission coupled into the lasing mode: 
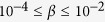
, which gives an average threshold value for the photon number^12^ 
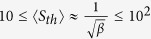
 and rather large relative fluctuations (assuming a Poisson distribution) 
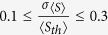
.

Under such conditions, we expect the transition to coherence to match the physical criteria of the mesoscale, where *phase-transitions* are known to become gradually smoother upon reduction of system size while retaining all the principal physical features (as is well known, e.g., in antiferromagnetic spin chains^13^, atomic clustering^14^, optical formation of atomic gratings^15^, or liquid systems^16^). Thus, studying a mesoscale laser will not only provide a characterization of its transition towards coherence, but also allow us to identify the *emergent features* of nanoscale devices.

## Results

Our experiment is performed in a small-sized VCSEL cavity (for details see Supplementary Information, sections I to IV, i.e., SI-I to SI-IV) with an estimated 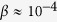, which corresponds to a threshold power emission 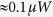 (sufficient for detection). As a result of this choice in cavity size, four indicators can be measured: (a) the full temporal dynamics, which allows for the computation of (b) autocorrelation, and (c) correlation functions, and (d) for the recontruction of the phase space (cf. SI-V and SI-IX). These indicators enable us to follow the buildup of the lasing emission throughout the threshold region.

The laser response to the current injection is shown in Fig. 2, which displays the usual S-shaped behaviour, typical of threshold crossing. The shaded part of the curve highlights the experimental region of interest. Figure 3 shows sections of the temporal traces corresponding to different points in the input-output response (Fig. 2) and to significant points of the autocorrelation (Fig. 4).

The time traces clearly show a qualitative change in the dynamics of the laser intensity as the pump is increased. The two bottom traces (grey and blue) – lowest pump values in the S-shaped curve (Fig. 2) – clearly show an intensity dynamics which is characterized by pulses superimposed on a background (partly due to technical noise), while the upper ones (red and black) display noisy oscillations. Additional information on these two clearly differing dynamical regimes can be gathered by looking at the autocorrelation function of the digitized signal, the standard technique used to estimate the degree of coherence of the radiation emitted by nanolasers^2^,^3^,^4^,^5^,^6^.

Figure 4 shows the autocorrelation 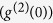 as a function of pumping current. Aside from the first two points, where the limited bandwidth of the measurement chain filters too strongly a very weak signal, two clearly differing regimes are recognized: one with a fast drop of the autocorrelation (until 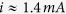) and a second one, with slope at least one order of magnitude smaller, which lasts until 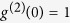 (i.e. the *Poisson limit*, reached, within the error bars, only at 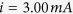 – cf. SI-V).

Previous observations of a double slope in the dependence of the autocorrelation on the pump have been reported in nanolasers^3^,^4^, while a slow convergence in the correlation (above threshold) was found in a VCSEL^17^ (but no fast drop in 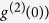. The slow convergence was later shown^18^ to be due to the Relaxation Oscillations (ROs). The absence of additional indicators in those papers make a close comparison with our results difficult, but it is plausible to consider them in agreement with our experiment.

A closer analysis of the laser output features can be carried out on the basis of the entire correlation function. Figure 5 shows the decay of the correlation for 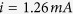 (grey line) accompanied by the hint of an oscillation with a delay time *τ* approaching 2*ns*. Although very small, the bump is significant, since the size of the error bar (not marked) is approximately four times smaller than the height of its peak. Its presence hints therefore at the existence of a small amount of correlation in the spikes (Fig. 3, grey curve) over this time scale (cf. radiofrequency – rf – power spectrum in SI-VI). More noticeable is the fact that 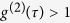 over the whole interval explored. This can be interpreted as a sign that the laser radiation is not yet in a fully coherent state, even though the drop-off appears to be slower than what is typically observed for incoherent (or chaotic) light^19^. The peak becomes rapidly more pronounced (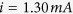, blue curve) at shorter delay time *τ*, but the correlation remains significantly larger than 1 (i.e., within error bar).

Crossing the break point in the autocorrelation function (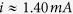, Fig. 4) the shape of 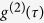 changes entirely: besides a noticeable reduction in its value at the origin 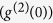, well-developed oscillations appear (red curve in Fig. 5; 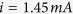), which match the rf power spectrum (cf. SI-VI) and continue beyond 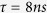. Most importantly, the oscillating correlation function periodically takes values 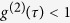 for given intervals of the time delay *τ*. This is the unequivocal sign of a change in the nature of the signal, by now coherent. Indeed, 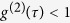 stems from the coherent oscillations between carriers and photons^18^. The oscillations in 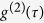 persist in the same form and gradually weaken to give rise, eventually, to a flat correlation function, with 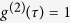 at 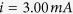 (black curve in Fig. 5).

A contradiction seems to surface here: if coherence is achieved only in the Poisson limit (at 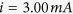), then the e.m. field should not be coherent at 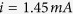. This discrepancy stems from the assumption that 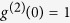, defined in Class A lasers as the onset of coherence, should hold for Class B devices. Instead, these more complex systems possess coherent oscillations between e.m. field and population (ROs) which necessarily produce 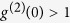. Thus, the value of 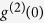 is inadequate for characterizing the e.m. field’s coherence in class B lasers which require, rather, the measurement of rf spectra (cf. SI-VI) and/or information on the full dynamics.

The existence of these two regimes of coherence, the first partially developing, the second fully established, but both identified by 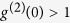, demonstrates the existence of new physics in the laser threshold region and explains the puzzling results obtained in nanolasers^2^,^3^,^4^,^5^,^6^, where the pulsed laser operation and extremely low power levels could hardly provide the complete information available in our experiment.

It is important to remark that dynamical intensity spiking has been reported in the emission of a near-infrared VCSEL emitting in two competing polarization states^20^, with correlation functions exhibiting oscillations similar to those we show in Fig. 5. There, the spiking output was correctly attributed to (and modelled as) the competition between the two polarization components. Although a close comparison between our results and those of ref. 20 cannot be drawn, it is possible that the *physical origin of the spikes* may lie in the physics of the coherence buildup even in those observations^20^ and that only the *anticorrelation between spikes* be due to the competition between the two polarization modes.

## Numerical Results

A recently developed Stochastic Simulator (SS)^21^ (details in SI-VII) offers the possibility of comparing the experimental results to numerical predictions which are solely based on the physics of lasing. The autocorrelation, 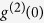, computed for 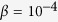 and for pump values compatible with those of the experiment (cf. SI-VIII) displays qualitative features in agreement with the experiment: a fast drop in the autocorrelation, followed by two plateaus and a slow decrease to the Poisson limit (Fig. 6). Despite the differences in magnitude for 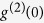 (sensitive to external noise) and in the pump values – the SS does not take into account the physics of semiconductors –, we remark that the extrapolation of the fast drop to 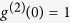 occurs for relative values above threshold which are quite close to the experimental ones (compare upper horizontal scales in Figs 4 and 6). The dynamical signal and the rf power spectra obtained from the SS agree qualitatively quite well with the main features displayed in Fig. 3 (spiking, oscillations, etc., Fig. 7). This is a strong, additional confirmation of our interpretation that the nature of the dynamics controls the value of 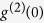.

A phase-space illustration^22^ of a section of the dynamics, in the spiking and oscillatory regimes, offers an additional tool for visualizing the laser’s behaviour in the transition region. Figure 7 shows a phase space representation of the temporal evolution of the laser field intensity at two different values of injected current. The two top panels show the experimentally reconstructed phase space, in the horizontal plane, while the bottom ones show the equivalent reconstruction obtained from a SS^21^. The depicted trajectory displays a short section of the temporal sequence taken from the long data ensemble from which the flow (horizontal plane) is reconstructed. Time is colour-coded for illustration and the values given in the bar correspond to time steps (0.1 *ns*). The vector field is computed as the average flow obtained from the noise-driven evolution of the trajectory (cf. SI-IX). The in-plane variables are chosen to represent a 2-D embedding of the laser dynamics^23^. Panel (b) clearly shows that the fixed point corresponding to the steady-state operation is a stable focus^24^, surrounded by the characteristic circulation of the flow (green arrows). The same feature is obtained by the SS (d), which offers an even better resolution than the experimental data (cf. SI-IX). This regime of operation clearly matches the above-threshold emission and optical coherence. At low pumping current (a), the flow takes an asymmetric shape concentrated along a band centered around the zero for the time derivative and little rotation. A much smaller degree of rotation in the flow is also found in the traces computed by the SS (c). The temporal trajectories, which represent the motion in the *average landscape* depicted by the flow, clearly illustrate the different evolution. While at large pump the motion corresponds to a (noise-driven, thus irregular) oscillation (panels b and d), at low pump isolated spikes clearly appear, connected by low-intensity dynamics (panels a and c). Since the SS is based exclusively on the fundamental physics of lasing, the appearence of spikes in its predictions can only be attributed to the stochastic nature of the physical process. This allows us to attribute the spiking (panel a) to the fundamental physics of the interplay of spontaneous and stimulated emission in Class B (mesoscale) lasers rather than to sources of technical noise.

## Discussion

A direct physical interpretation is possible on the basis of the time traces and of the picture offered by the SS^21^: the stochastic nature of the emission process and its random repartition among the e.m. cavity modes leads to a temporally non-uniform presence of (spontaneous) photons in the laser mode. When the number of photons is small, it is reasonable to expect (cf. SI-XI) that there will be time intervals with no photons on-axis. Since the seed for stimulated emission comes from the spontaneous photons, there will also be times when no coherent emission can take place. On the other hand, since transmission through the output mirror is also a stochastic process at the single photon level, an accumulation of energy beyond the equilibrium value may, at times, take place in the form of excess excited *dipoles* (carriers, atoms, etc.). Release of this energy takes place in the form of a pulse, as in any *dumped cavity* (e.g., Q-switched), thus explaining the presence of individual spikes in the laser output. Hence, we physically understand the origin of the emission bursts which are observed in the experiment and which lead to a *new kind of threshold dynamics* and to *anomalous behaviour in the autocorrelation functions*, which so far have been proven hard to interpret for nanolasers. This pulsed regime may well coincide with the observations of laser emission with a high degree of coherence over short coherence times recently reported in nanolasers^19^, but this point needs further confirmation.

Theoretical support for these considerations can be obtained from a simple topological analysis of Class B lasers, where a stability analysis performed just above threshold shows that the eigenvalues corresponding to the eigendirections in phase space are of the same order of magnitude (cf. SI-X). Thus, it follows immediately that the trajectories should evolve in a plane, as shown in the experimental and numerical reconstructions (Fig. 7), rather than along a unidimensional manifold, as in a Class A laser.

The combined evidence resulting from the different experimental indicators, and supported by numerical and analytical modeling, support the following conclusion: in a Class B mesoscale laser, the laser field builds up gradually in the form of individual light pulses whose repetition rate increases until fusion into an irregularly oscillating signal. The signal’s autocorrelation decreases rapidly during this phase. The onset of (irregular) oscillations corresponds to a regime where the laser field is fully developed, thus *phase coherent*. Hence, the residual, slow evolution of the autocorrelation towards the Poissonian limit does not signify imperfect coherence due to the combination with residual spontaneous emission, but rather stochastically-induced dynamics between coherent photons and carriers. Taking the small peak in the delayed correlation (for 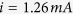) as a sign of the *onset of mutual correlation between pulses*, we quantitatively show that the fully correlated regime (i.e., noise-induced relaxation oscillations) occurs much sooner, with respect to this reference (*i*_*R*_), than the point at which the Poisson limit 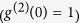 is reached.

**Figure 1:**
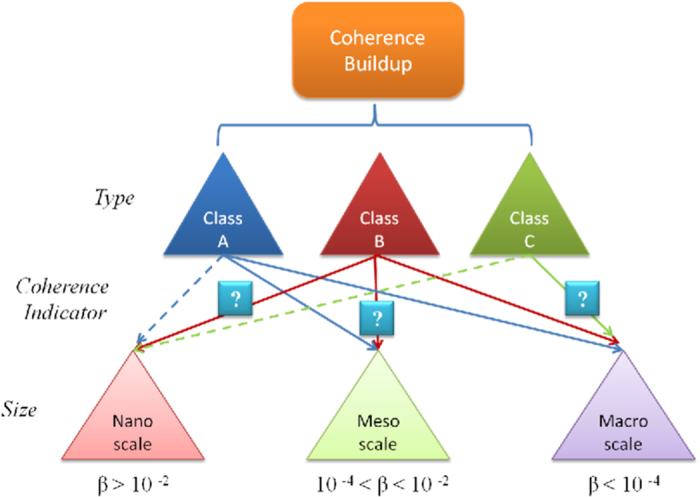
Transition to coherence according to dynamical class and cavity size, as characterized by the value of the spontaneous emission fraction parameter, *β*. Color coded, solid lines linking a given class with a cavity size indicate existing lasers. Dashed lines connect dynamical classes to potentially realizable cavity sizes. Superimposed question marks denote transitions to coherence in need of investigation. Recent work^29^ has confirmed the known photon statistics in a Class A VECSEL (considered a mesoscale device here). Nanolasers with extremely high cavity quality factor may become Class A and may be expected to follow the standard photon statistics. Indirect evidence^25^,^26^ hints to similarities between the statistical behaviour of macroscopic Class B lasers and Class A, but no final proof is available so far. Finally, no work exists for Class C lasers (mostly large-sized, Far-InfraRed lasers); current theoretical predictions of Class C-like operation in nanolasers^8^ do not yet find experimental verification.

**Figure 2:**
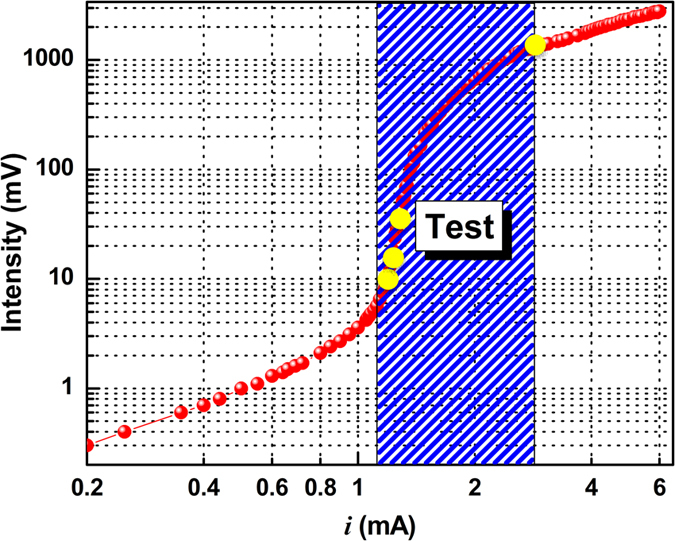
Output intensity as a function of injection current. The blue-shaded area highlights the region where measurements are taken. The yellow dots correspond to the sample measurements displayed in Fig. 3.

**Figure 3:**
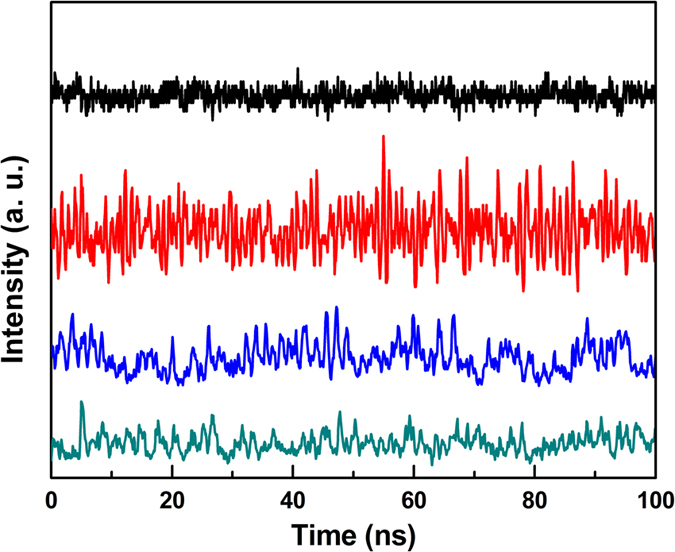
Intensity fluctuations as a function of time for different injection current values (grey, blue, red and black lines represent *i* = (1.26, 1.30, 1.45, 3.00) *mA*, respectively). The measurement points are marked on the laser response curve with yellow dots (Fig. 2). The curves are vertically shifted for clarity.

**Figure 4:**
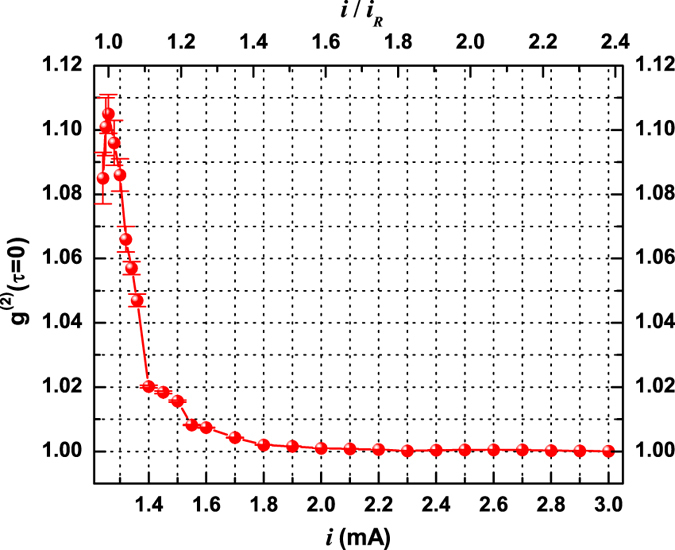
Measured second order autocorrelation (*g*^(2)^(*τ* = 0)) as a function of pumping current. The fast drop in the autocorrelation, if *extrapolated* to the Poissonian limit, would predict full coherence at 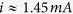, i.e., approximately at 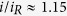 (cf. upper horizontal scale, where 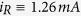 – the level for which a small peak in the delayed correlation function appears, Fig. 5, grey line). On the other hand, 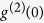 reaches the Poisson limit only at 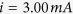, i.e., 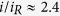.

**Figure 5:**
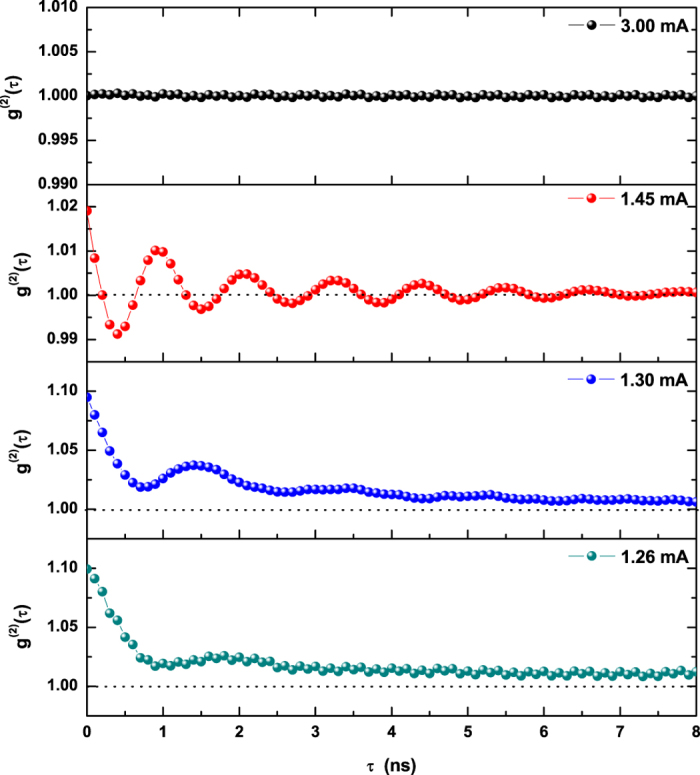
Second-order correlation function *g*^(2)^(*τ*) for the laser injection current values matching those ofFig. 3. In the top two panels the delayed correlation, computed in the range where the autocorrelation is decaying fast, never goes below 1 (Poisson limit). The negative parts of 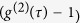 in the third panel (red line) correspond to coherent oscillations of carriers and field intensity and therefore signal the presence of full coherence in the field.

**Figure 6:**
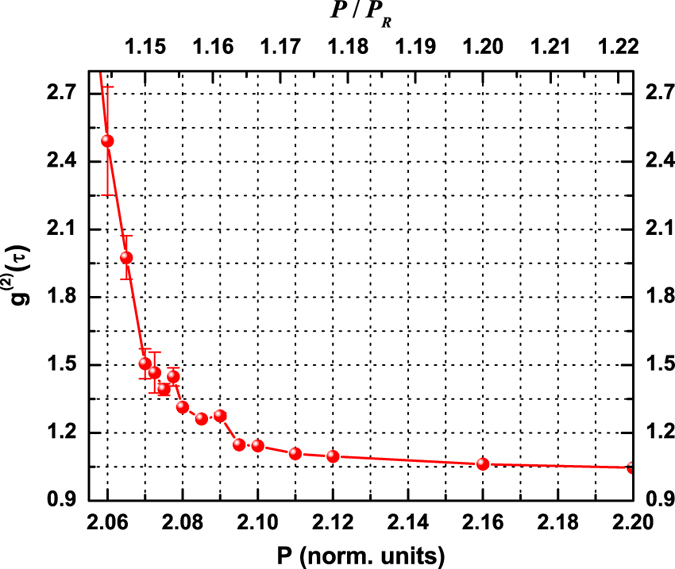
Computed second order autocorrelation (*g*^(2)^(*τ* = 0)) on the laser dynamics forecasted by the SS^21^ as a function of pump. Extrapolation of the fast drop in autocorrelation to the Poisson limit yields a relative pump value 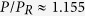 in good agreement with the experiment (cf. Fig. 4 and SI-VIII for details).

**Figure 7:**
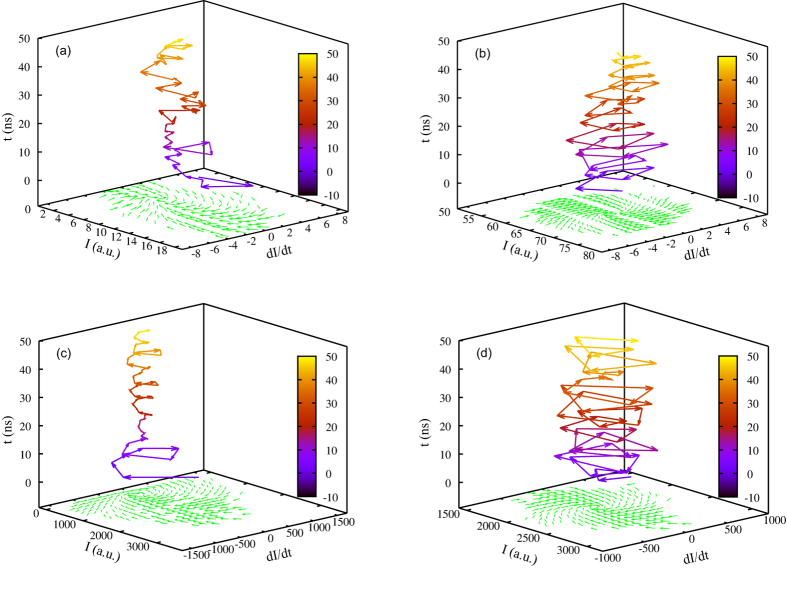
Flow (green arrows in horizontal plane) and temporal trajectory (in color) for different experimental pump current values – (a) *i* = 1.26 *mA* and (b) *i* = 3.00 *mA* –, and for corresponding normalized pump values – (c) *P* = 2.5 and (d) *P* = 3.0 – obtained from a Stochastic Simulator^21^. The short sections of temporal evolution, extracted from the long datasets used for computing the average flow, illustrate the typical laser emission in the different pump regimes. 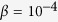 for the simulations (compatible with the estimated spontaneous emission fraction for the experimental device, cf. SM-III). No external noise sources are included in the SS, thus the stochastic behaviour is entirely due to the physical processes giving rise to lasing. The flow is reconstructed using a 2-D embedding consisting of the output intensity and its time derivative (cf. SM-IX for details). The scales are normalized. Two entirely different kinds of evolution are identified: one at very low pumping (panels (**a**,**c**)), the other at large pumping levels (panels (**b**,**d**)). The time scale is given in points (time step 0.1*ns*), while the intensity and intensity’s time derivative scales are in *mV* and *mV*/*point*, respectively.

The choice of a mesoscale laser proves crucial for reaching these conclusions since its reduced cavity volume allows for a transition region which is sufficiently wide to ensure parameter stability. While numerical simulations employing the SS^21^ predict transition features similar to the observed ones in all Class B lasers, macroscopic, low-*β* cavities are reasonably well characterized by a very sharp threshold (cf., e.g., S-curve for 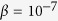 – Fig. 2 in^21^). Hence, while present, the dynamics may prove to be experimentally inaccessible since intrinsic parameter fluctuations would make it impossible to ensure stable operation at fixed pump, thus masking the intrinsic dynamics with noise-induced jumps between the lower and the upper branches of laser operation. This constraint explains how threshold statistics studies have failed to detect discrepancies between observations in macroscopic Class B lasers and the traditional Class A description (Fig. 1 and^25^,^26^).

In reference to Fig. 1, our results allow for advances in the characterization of the transition to coherence in all Class B lasers: their extrapolation on threshold dynamics and on the development of field coherence to nanolasers is supported by numerical evidence provided by the Stochastic Simulator (cf. SI-IX) which predicts the persistence of the light spikes for small cavities. Independently, short coherence times have been measured^19^, and predicted^27^, in a nanolaser experiment which unequivocally shows that the statistical superposition state between spontaneous and stimulated emission predicted by Class A coherence theory is incompatible with *Class B* dynamics. Since only cross-correlation functions were measured there^19^, it is not possible to establish a direct relation to our results, but it is plausible to expect that the light spikes may be the source of the observation of good quality correlation, on short times. Work is ongoing to elucidate this point. Note, however, that since, so-far, all nanolasers are Class B devices, some confusion reigns between the dynamical features due to their parameter values – laser class – and their cavity size. This investigation unequivocally clarifies this point.

In perspective, while some fundamental issues related to spontaneous and stimulated emission in subwavelength cavities^10^ remain still open, our results clearly show how some of the inconsistencies encountered in the interpretation of experiments performed in micro- or nanolasers^2^,^3^,^4^,^5^,^6^,^7^ are simply due to a misuse of theoretical predictions which hold only for Class A systems. Our experimental results also support the statement that no clear “threshold” value can be assigned for mesoscale lasers (and *a fortiori* for nanolasers), in agreement with current knowledge on the physics of “phase transitions” in small systems.

Finally, our observations open new paths for a clear picture of the establishment of the lasing process in small systems, where the discreteness in the number of *dipoles* and photons plays a crucial role: in addition to the issues related to coherence buildup, the presence of light spikes may be deleterious for numerous applications, e.g. in information processing, on-chip data transmission, biomedical application, etc. On the other hand, the natural occurrence of this regime may also pave the way to new applications, not previously conceivable.

## Methods

Data are sampled at 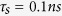 and acquired in single shot with 10^6^ points per sequence, thereby minimizing parameter drifts (measurement time 0.1*ms*). Second order correlation functions are computed for the measured intensity *I*(*t*) in the standard way^4^: 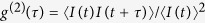 where 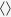 represents time average and *τ* is the delay time (autocorrelation corresponds to *τ* = 0). The phase space reconstruction is obtained with an embedding technique^24^ using the laser intensity and its derivative (dynamically equivalent to the number of excited dipoles^28^).

## Additional Information

**How to cite this article**: Wang, T. *et al.* Dynamical Buildup of Lasing in Mesoscale Devices. *Sci. Rep.* **5**, 15858; doi: 10.1038/srep15858 (2015).

## Supplementary Material

Supplementary Information
