# Supplementary material for: Dynamical Buildup of Lasing in Mesoscale Devices
Source: Sci Rep. 2015 Oct 29;5:15858. doi: 10.1038/srep15858 (PMC4625369; doi:10.1038/srep15858)
Supplement: Supplementary Information [file srep15858-s1.pdf]

Dynamical Buildup of Lasing in Mesoscale Devices
Supplementary Information

T. Wang[1,2], G.P. Puccioni[3], and G.L. Lippi,[1,2]
[1] *Institut Non Linéaire de Nice, Université de Nice-Sophia Antipolis*
[2] *UMR 7335 CNRS, 1361 Route des Lucioles, F-06560 Valbonne, France and*
[3] *Istituto dei Sistemi Complessi, CNR, Via Madonna del Piano 10, I-50019 Sesto Fiorentino, Italy*

PACS numbers:

I. EXPERIMENTAL SETUP

The experimental setup is shown in Fig. 1 and consists of a commercial VCSEL (Thorlabs VCSEL-980) with maximum allowed pump current $i_{max} = 10mA$ for a maximum output power $P_{max} \approx 1.85mW$ (manufacturer's specifications), mounted on a TEC module (Thorlabs TCLDM9) which allows for temperature stabilization from an external source. The current is supplied by a commercial dc power supply (Thorlabs LDC200VCSEL) with resolution 1 μA and accuracy $\pm$ 20 μA. The current drift at constant ambient temperature is less than 1μA over 30 minutes and both noise and ripple are smaller than $1.5\mu A$ (manufacturer's specifications [1]). The laboratory's temperature is stabilized at $(20 \pm 1)°C$ while the laser temperature is set at $T_L = (25.0 \pm 0.1)°C$. The active temperature stabilization comes from a home-built apparatus, capable of stabilizing to better than $0.1°C$. In the current range investigated, the laser emits on a single linear polarization, devoid of switching.

The signal output is passed through an optical isolator consisting of a Polarizing Beam Splitter (PBS) (Thorlabs PBS053), with extinction ratio ($T_p : T_s = 1000 : 1$), followed by a zero-th order, $\lambda = 980nm$ Quarter-Wave-Plate (Thorlabs WPQ05M-980 – retardance accuracy $< \lambda/300$, residual reflectance $< 0.25\%$ per surface). The signal is then coupled, through its multimode optical fiber (diameter $d_f = 62.5\mu m$), into a fast, amplified photodetector (Thorlabs PDA8GS) with bandwidth $9.5GHz$. The electrical signal is digitized by a LeCroy Wave Master 8600A oscilloscope with $6GHz$ analog bandwidth and up to 5×10^6 sampled points per trace. The data are stored in a computer through a GPIB interface using Python.

The detector is supplied by a dc 12V battery, rather than by the power supply provided by the Manufacturer, to reduce the influence of electrical noise. Special care is taken to minimize all external electrical noise sources, thus the leads carrying the dc current from the battery to the detector are shielded and grounded. Ground loops are avoided in the detection section and a single high quality ground is used for the whole electrical acquisition chain. Nonetheless, some residual electrical noise is present with some spectral components around 3 GHz.

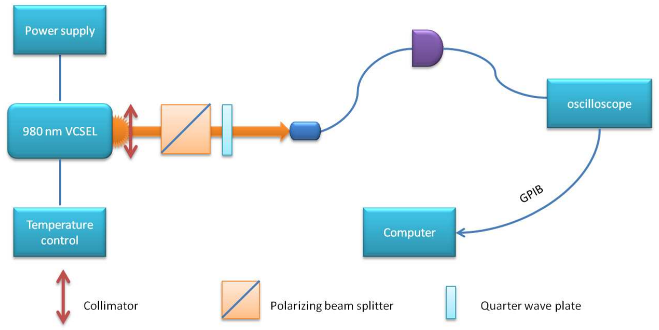

FIG. 1: Experimental setup. The bottom part of the graph illustratest the three optical elements interposed on the path between the laser and the detector. The VCSEL is supplied with a stabilized current source and is temperature-controlled. The digitized intensity sequence is stored in a computer for post-treatment.

II. MEASUREMENT TECHNIQUES

Acquiring large datasets of the laser's output signal at high rates presents several advantages. Indeed, the stored data can be used not only for computing correlation functions (in principle even of order higher than two), but also for checking the laser's radiofrequency spectrum, the shape of the temporal output (thus its dynamics) and its phase space representation.

All data used in this paper are sampled at $\tau_s = 0.1ns$ and sets contaning 10^6 points are acquired in single shot and stored. This avoids external drifts, since the full measurement time is $t_m = 0.1ms$. The detector's offset, particularly important for low level signals, is determined by acquiring one dataset in the absence of optical signal for each group of measurements (i.e., every time the current is changed). An average is taken from the acquired offset signal and is subtracted from all the other datasets. Notice that this operation may, in principle, produce slightly negative electrical signals – representing the laser power – when the latter is so low as to be negligible. Indeed, since we subtract the average, a fluctuation may result in an apparently negative power value. However, since the data is acquired with finite resolution (8 bit) restoring the original resolution (together with correct rounding of the results) avoids almost all false negative values.

In order to compute the correlation functions, we split

the single trace (with 10^6 points) into ten traces of equal length. On these, we compute in MATLAB® the second-order correlation function $g^{(2)}(\tau)$ with a time shift $0 \leq \tau \leq 1000 \times \tau_s$. The 10 replicas for each experimental condition (current value) are used to compute the average and standard deviation shown in the autocorrelation figures.

For the average output measurements (S-curve, Fig. 2b) we use a BPW34 PIN silicon photodiode with spectral sensitivity at $S(\lambda = 980) \approx 0.4 A/W$, suitably amplified (with low bandpass) to obtain sufficient signal in the spontaneous emission region (cf. Fig.2b). The measured output is taken with a multimeter with sub-mV resolution.

Fast measurements are taken with the Thorlabs PDA8GS, whose photodiode possesses a spectral responsivity $S(\lambda = 980nm) \approx 0.75 A/W$; after the internal stage of amplification, built-into the detector ensemble, and coupling on $R_{in} = 50\Omega$ we obtain in output a $V_{out} = 1mV$ signal when the input power is $W_{min} \approx 3\mu W$ [2], which represents the smallest fast signal we can resolve. Converting into photon number at $\lambda = 980nm$, this minimal signal corresponds to $N_{phot} = 1.6 \times 10^{13}$, which scaled to the cavity lifetime (estimated to be $\mathcal{K} \approx 10^{11} s^{-1}$) gives 160 photons *intracavity*. This value matches in order of magnitude the number of photons at threshold. Assuming a mirror transmissivity $T \approx 0.1\%$, this corresponds to a fraction of one photon exiting the cavity in a time $1/\mathcal{K}$. Since the detector bandwidth is approximately ten times smaller, this corresponds to $< N_{ph,det} >_{th} \approx \mathcal{O}(10^0)$. Thus, we see that the fast detector cannot resolve the average photon number at threshold, but will be able to dynamically measure photon spikes whose amplitude is about one to two orders of magnitude larger. These estimates are consistent with the observations which show spikes a few times larger than the background (Fig. 3 of paper, bottom trace), while the simulation obtained from a model based on laser principles show a much better contrast (cf. Section XI).

III. ESTIMATE OF THE VALUE OF β

Extrapolation of the two straight lines in the input-output curves plotted in log-log scale allows for an estimate of the value of β [3]. We can independently check the validity of this extrapolation by comparing computed estimates using the Manufacturer's Specifications [4] and standard modeling equations [5].

A. Experimental determination of β by extrapolation

A direct estimate of the value of β can be obtained from the extrapolated curves of Fig. 2b. Supposing for simplicity the Purcell factor [6] to be negligible (cavity Q-factor $\approx 15 \times 10^3$, obtained from cavity losses $\mathcal{K} \approx 10^{11} s^{-1}$, L_c from Table I and reflectivity of the outcoupling VCSEL mirror $R \approx 99.9\%$ [7]), we can assume the spontaneous emission distribution to be spatially homogeneous. Thus, defining the optical collection angle Θ_c seen by the detector through the collimator (cf. Fig. 2a), the ratio between the actual number, N_{sp}, and the collected number, N_c, of spontaneously emitted photons is

$$\frac{N_{sp}}{N_c} = \frac{4\pi}{\Theta_c}. \quad (1)$$

Estimating $\Theta_c \approx \pi\alpha^2$, where α ($\approx 20°$) is the angle characterizing the aperture of the collimator, we find $\Theta_c \approx 0.38 sr$ and $\frac{N_{sp}}{N_c} \approx 0.03$. From Fig. 2b we obtain $F \approx 10^{-2}$, which combined with the previous ratio gives an estimate

$$\beta \approx F \frac{N_{sp}}{N_c} \approx 3 \times 10^{-4}. \quad (2)$$

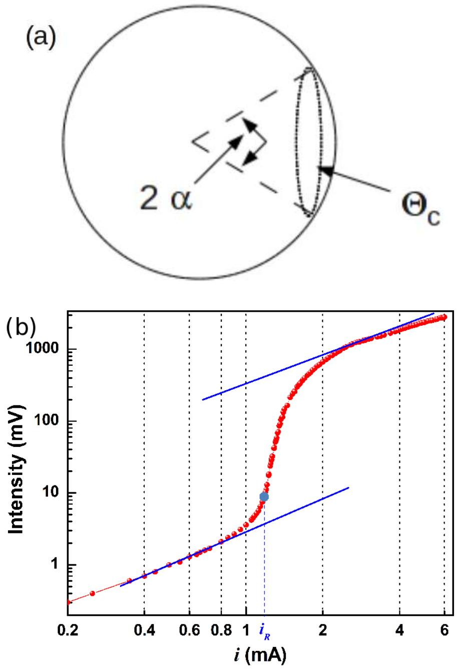

FIG. 2: (a) Schematics of the collection angle for the spontaneous emission: the collimator collects light in the solid angle Θ_c, defined by the aperture of the collimator (α). (b) Output vs. input laser response in logarithmic scale with extrapolated lines. The ratio F between coherent output and spontaneous emission is estimated using the intersections between the vertical line and the prolongations of the two branches. The blue dot marks the reference point used in the text for normalizing the upper axis of Fig. 4 and matches the corresponding point in Fig. 4 (section VIII).

B. Estimating the cavity's diameter from Manufacturer's data

The typical beam divergence (FWHM) specified by the Thorlabs catalogue is $2\theta = 25°$, defined for the $\frac{1}{e^2}$ point of the intensity. Assuming a Gaussian beam of waist w_0, we can use the far-field divergence θ_{ff} [8] of the beam to obtain an estimate of the beam radius

$$w_0 = \frac{\lambda}{\pi \theta_{ff}} \,. \tag{3}$$

Since θ_{ff}, eq. (3), is defined at the $\frac{1}{e}$ point of the field amplitude and θ used by the Manufacturer is defined at the $\frac{1}{e^2}$ point of the intensity, we can use $\frac{\theta}{2}$ in place of θ_{ff} to find

$$w_0 = 1.4 \mu m \,. \tag{4}$$

The field intensity decreases to $\approx 14\%$ over a distance equal to w_0 and to about 3% at $2w_0$. Thus it is reasonable to set an expected radius for the cavity boundary $r_c \approx 2w_0$. This way, we estimate the cavity diameter $d \approx 6\mu m$ using the information from the Manufacturer's datasheet. In order to calculate the cavity volume, we need an estimate of the effective cavity length, as discussed in the following subsection.

C. Theoretical estimate of β

An effective cavity length can be defined using [9]

$$L_c = \frac{\lambda}{2} + 2L_{eff} \,, \tag{5}$$

$$L_{eff} = \frac{\lambda}{4\Delta n} \tag{6}$$

where L_{eff} is the penetration depth into the Bragg reflectors (assumed symmetric on the two sides). Thus the cavity volume becomes

$$V_c = \frac{\pi d^2}{4} \left[\frac{\lambda}{2} + 2L_{eff} \right] \,. \tag{7}$$

A standard theoretical expression (eq. (9) in [5]) for estimating the β parameter (fraction of spontaneous emission coupled into the lasing mode) reads

$$\beta = \frac{3}{2} \frac{\Gamma K \lambda^4}{4\pi^2 n^3 V_c \Delta\lambda_s} \,. \tag{8}$$

Using the values for the constants given in Table I, we obtain

$$\beta \approx 1.8 \times 10^{-4} \,. \tag{9}$$

The two estimates, eqs. (2,9) give values which are quite close. Given that for each of the estimates we have made a number of hypotheses and approximations, we only retain the order of magnitude for the spontaneous emission factor and settle for $\beta \approx 10^{-4}$. This result places this laser near the lower limit for characterizing a device as mesoscale.

TABLE I: Parameters used for the estimate of β. Sources for the values are given in the third column.

Parameter	Value	Source
Γ	0.038	Ref. [9]
K	1	Ref. [5]
λ	$980nm$	Ref. [4]
n	3.6	Ref. [9]
V_c	$39\mu m^3$	eq. (7)
L_c	$1.37\mu m$	eq. (5)
$\Delta\lambda_s$	$4nm$	measured at $i = 1.20mA$
Δn	0.56	Ref. [10]

IV. STABILITY AND REPRODUCIBILITY

The pump interval where the autocorrelation function quickly decreases corresponds to a current range $\Delta i \approx 0.140mA$, thus the accuracy with which the current values can be set and held is important. According to the Manufacturer's specifications [1] each current value can be set within $\pm 20\mu A$ (cf. Section I). This accuracy produces an error in the placement of each point equal to 1/5 of the minimal division in Fig. 4 (main paper) – i.e., a horizontal error bar on the pump current (not shown).

From the ripple and noise Manufacturer's specifications (cf. Section I) it is straightforward to see that the perturbation on each current value is less than 7% of each set point. Thus, even though in Fig. 4 (main paper) the slope in the first decay region is rather steep, nonetheless the current stability is sufficiently high to ensure that each acquired data stream is consistently taken at the same pump value (at least from the power supply's stability).

V. MEASUREMENT OF THE CORRELATION FUNCTION

The probability distribution for obtaining a fluctuation at a shifted time τ, correlated with a fluctuation at any given instant, starts at $g^{(2)}(0) = 2$ below threshold, but this value can be measured only by instrumentation with a bandwidth larger than the shortest physical time scale. This is virtually impossible when dealing with semiconductor-based devices, since the fastest time scales are in the range of the picosecond. The maximum value of the correlation which can be measured, with limited bandwidth, is therefore

$$g^{(2)}(0) \approx 1 + \frac{\tau_{det}}{\tau_c} \,, \tag{10}$$

where τ_{det} represents the characteristic time constant of the detector, while τ_c denotes the photon's lifetime in the cavity (since a spontaneous photong will, in average, remain inside the cavity for a time of the order of τ_c).

Fig. 4 (main text) shows the autocorrelation as a function of pump (current). The first two points ($i = 1.22mA$ and $1.24mA$) show an increase of the autocorrelation due to the limited bandwidth of the measurement chain (eq. (10)), where we estimate $\tau_{det} \approx 0.1ns$ and $\tau_c \approx 10ps$ on the basis of standard cavity parameters (cf. Section III A).

The amplitude of the fluctuations is fairly large near the correlation peak (error bars), but decreases quite rapidly as the autocorrelation value drops down, until the bars become invisible on this scale (at approximately $i \approx 1.5mA$). Although not resolved in Fig. 4 (main text), the value $g^{(2)}(0) = 1$, i.e., the Poisson limit, is not reached within the error bar until the end of the range shown in the figure. The slow convergence is explained in the main text.

VI. RELAXATION OSCILLATIONS AND SPECTRA

As already suggested by the correlation functions (Fig. 5 – main text), Relaxation Oscillations (ROs) accompany the evolution of the e.m. field growth throughout. Although a full discussion goes beyond the scope of this presentation, it is interesting to remark that qualitatively different spectra are associated to the different $g^{(2)}(0)$ features (a. large slope, b. plateaus of different value, c. convergence to the Poisson limit). For low pump ($i = 1.26mA$), the rf spectrum is broad and decreases almost monotonically with frequency, with only a hint of a shoulder around $f = 0.5GHz$ (Fig. 3 – top left panel). When the autocorrelation changes its slope abruptly ($i = 1.40mA$, Fig. 4 – main text) a well-developed peak is visible at frequency slightly below $1GHz$, but the low-frequency component is just as large (Fig. 3 – top right panel). At the next plateau ($i = 1.60mA$, Fig. 4 – main text) the rf spectrum presents a large, sharp peak just above $1GHz$ (Fig. 3 – bottom left panel). Increasing the pumping current even further, produces a broader and shallower peak(Fig. 3 – bottom right panel), as is well-known for ROs in VCSELs [9].

VII. STOCHASTIC SIMULATOR

The Stochastic Simulator (SS) [13] has been employed to obtain predictions for the dynamics of a mesoscale laser in the transition region leading to lasing action. The parameters values used for the simulations are: spontaneous emission fraction $\beta = 10^{-4}$, carrier density relaxation rate $\gamma = 2.5 \times 10^9 s^{-1}$, cavity relaxation rate $\Gamma_c = 1 \times 10^{11} s^{-1}$, relaxation rate for off-axis spontaneous photons $\Gamma_o = 1 \times 10^{13} s^{-1}$. Since the Simulator is based on physical principles (coupling between radiation and ensemble of emitting *dipoles* [13]), rather than on semiconductor physics, the pump values have been chosen in a phenomenological way, so as to qualitatively match the experimental input-output curves (Fig. 2b, section III B) with the average laser output predicted by the SS (Fig. 4, section VIII).

In order to match the experimental conditions, since, as in [13], the time increment is very small ($\tau_{ss} = 2fs$), we have integrated the predicted output on a time window $t_D = 0.1ns$, compatible with the bandpass of the detector in the experiment. The time sequences thus obtained are treated in the same way as the experimental ones.

VIII. NUMERICAL $g^{(2)}(0)$

The numerical autocorrelation functions are computed on the output of the SS, low-pass filtered (on 32768 points per file) to simulate the detector's response, and averaged over 20 independent runs (starting from different noise seeds). Since the pump parameter is not comparable to the current input into the VCSEL, in order to compare the curves we have normalized the pump scale (P/P_R in the upper horizontal scale of Fig. 6 – main paper) in the same way as in the experiment.

The reference point for pump normalization (blue dot in Fig. 4) is the equivalent of the point at $i_R = 1.26mA$ (Fig. 2b, section III B). It is determined by placing the operating point on the average laser output at an ordinate above the extended lower branch taken in proportion to the experimental figure (Fig. 2b). The corresponding pump value, P_R, is then read off the graph. The computation must be carried out, however, remembering that while the numerical figure provides directly the value of β (from the ratio between the lower to the upper branch), the experimental one requires the correction discussed in Section III A (for this estimate here we have used a slightly more precise value for $F \approx 7.8 \times 10^{-3}$). The pump value for the best determination of the reference point is $P_R \approx 1.8$.

Notice that the procedure we have used is exclusively based on the experimental and numerical response curves, and makes no use of dynamical information. Thus, the surprisingly good consistency between the relative values at which the sharp drop in autocorrelation is observed both in the experiment (Fig. 4 – main text) and in the numerics (Fig. 6 – main text) is a confirmation of the soundness of our results.

IX. PHASE SPACE REPRESENTATION

The typical phase space representation is given in terms of laser intensity as a function of the carrier density, when analyzing data originating from a rate equations model [11]. However, an alternative, more experimentally accessible representation can be gained by using the measured intensity as a function of its time derivative [12]. The general features of the phase space are equivalent, even though some details will change (e.g.,

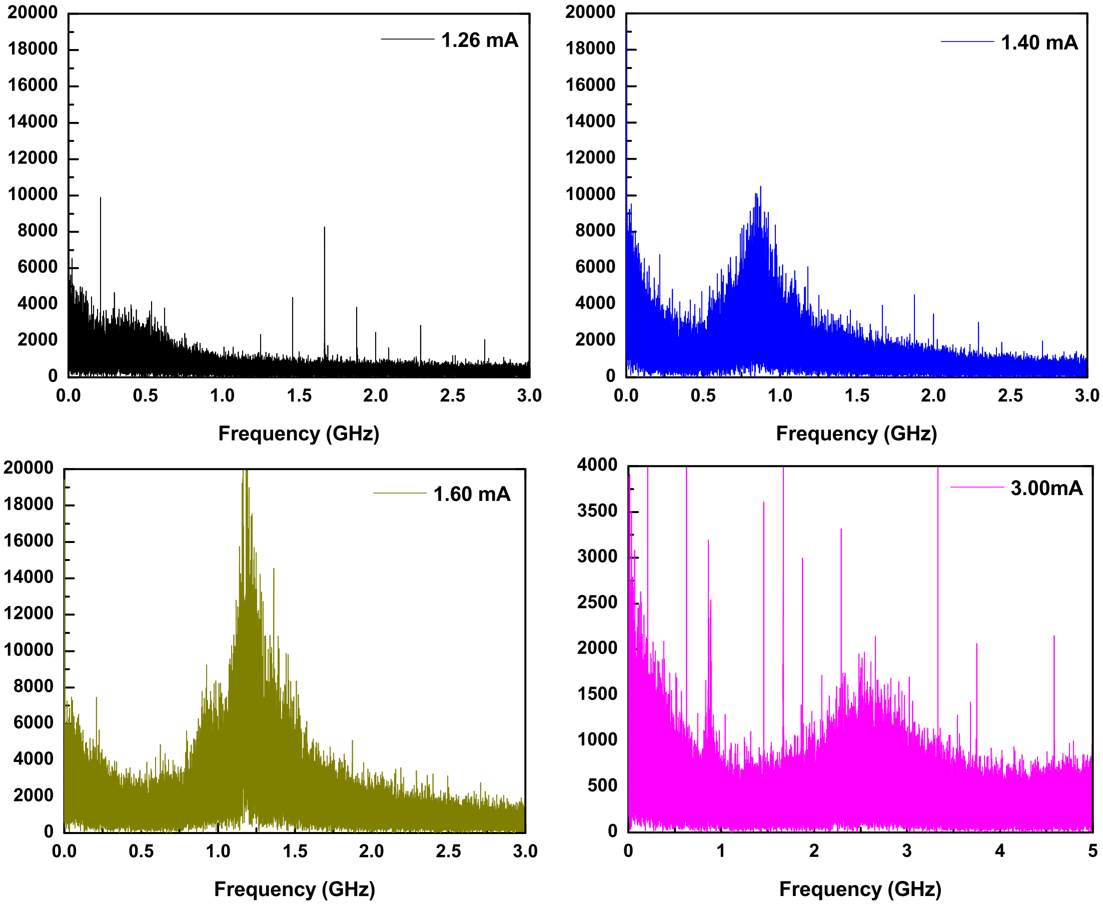

FIG. 3: Radiofrequency power spectrum for the experimental signal, computed on 10^6 points at different current values: $i = 1.26mA$ (top left); $i = 1.40mA$ (top right); $i = 1.60mA$ (bottom left). For $i > 1.60mA$ (bottom right, for $i = 3.00mA$) the resonance frequency shifts to higher values, the amplitude of the resonance is decreased and its width increased, as is well known from ROs theory [9].

the intensity's time derivative will be both positive and negative, while the carrier density is stricty positive).

Since the phase space representations used in the main article are obtained from temporal sequences, either experimental or numerical, the technique used to treat the data is the same. No modeling assumption is made in treating the data for Fig. 7 (main text).

The 3-D figures give a representation of the temporal evolution, color encoded along the vertical, time axis, of the field intensity plotted in phase space. A small section (< 50 points) of the temporal evolution is selected, as illustration of the dynamical evolution, from the complete (10^5 points) datafile. In the horizontal plane the average vector field representing the detected intensity and its time derivative are plotted. The vector field is obtained from the sequence of data points (experimental or numerical) by dividing the plane in rectangles in which the orientation of each vector is computed. From the ensemble of vectors, an average orientation is obtained and a standard length is assigned to each vector.

The reconstruction proceeds as follows. The time derivatives are computed for each time step using the following algorithm

$$\dot{I}_n = \frac{I_{n+1} - I_{n-1}}{2\tau_s}$$

and the corresponding $(2 \times (N-2))$ matrix is constructed (as for the temporal trajectory) – N being the total number of acquired points. After determining the extrema in both variables (i.e., I_{min}, I_{max}, $\dot{I}_{min}$, $\dot{I}_{max}$), an $M \times M$ phase space (S_{ph}) matrix is prepared ($M = 20$ in the figures) and all the measured points of the matrix are sorted to be assigned to an element of S_{ph}. Then, from the ensemble of vectors belonging to each element of S_{ph}, we compute the average vector which gives the best estimate of the direction of evolution of the trajectory. Notice that the number of points in each element of S_{ph} is **not** constant, thus the *outer* points in the matrix, with few occurrences, may be rather unreliable. The core elements, near the center of the matrix, carry the most reliable information and show the direction of the flow. The data are plotted with a rescaling factor, for ease of representation. Notice that in order to avoid large multiplicative factors in the scale, we have used $\tau_s = 1$ in the calcula-

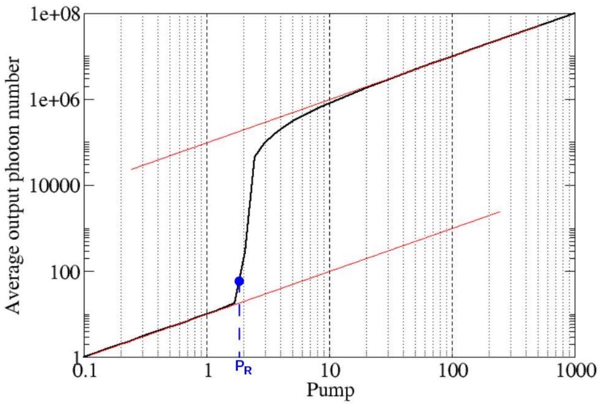

FIG. 4: Input-output curve predicted by the Stochastic Simulator for $\beta = 10^{-4}$ and parameter values given in the text. P_R represents the reference pump used for the normalization of the upper horizontal scale of Fig. 6 (main text) and matches the corresponding point (at $i_R = 1.26mA$) in Fig. 2b (section III B).

tions. We also remark that the discretization introduced by the sampling oscilloscope (cf. Section II) leaves some traces in the reconstructed flow (green arrows) recognizable through white bands, especially in panel (b) of Fig. 7 (main text).

X. STABILITY ANALYSIS

Using a standard model for a Class B laser (e.g., eqs. (2) in [11])

$$\dot{I} = K(D-1)I\,, \quad (11)$$
$$\dot{D} = \gamma(P - D - ID) \quad (12)$$

and studying the stability for the lasing solution

$$I_s = \; = P - 1\,, \quad (13)$$
$$D_s = 1\,, \quad (14)$$

we find the generic form for its eigenvalues

$$\lambda_\pm = \frac{-\gamma P \pm \sqrt{\gamma^2 P^2 - 4\gamma P(P-1)}}{2}\,. \quad (15)$$

It is straightforward to see that when $1 \leq P \lesssim 1 + \frac{\gamma}{4K}$ both eigenvalues are real and take values very close to each other. We can estimate their maximum difference by substituting the upper pump value of the range into eq. (15) to obtain

$$r \equiv \left| \frac{\lambda_+ - \lambda_-}{\lambda_+ + \lambda_-} \right|\,, \quad (16)$$
$$\approx \sqrt{\frac{1}{2}\frac{\gamma}{K}}\,. \quad (17)$$

Given that for a Class B laser the ratio $\frac{\gamma}{K} \ll 1$, this implies that the relative difference between the two eigenvalues, representing the two contracting eigendirections, is small and the dynamics takes place on comparable time scales. Explicitely, this implies that the dynamical evolution of the trajectory of the perturbed laser (e.g., passing threshold, but also perturbed by noise) must evolve in the plane represented by the two variables D (laser population inversion, or generically *excited dipole number*) and I (laser intensity, proportional to the photon number), cf. Fig. 7 (main paper). This feature clearly differentiates the threshold dynamics of a Class B laser [14], compared to a Class A one [15], where the unidimensional nature of the description forces the evolution along one single direction.

Specifically, for the parameters used in the computations, based on common VCSEL parameters, we obtain an estimate for $r \approx 0.1$, thus the maximum relative difference between the eigenvalues is of the order of 10% in the region of interest.

XI. SPIKE INTERPRETATION AND EXTRAPOLATION TO NANOLASERS

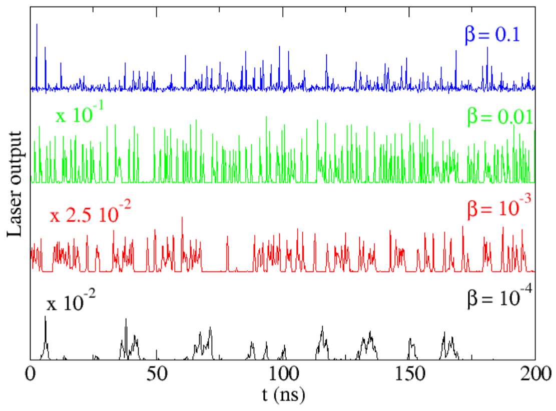

FIG. 5: Predictions of the total laser output (spontaneous plus stimulated emission) obtained from the Stochastic Simulator [13] for different fractions of spontaneous emission coupled into the lasing mode (β parameter). For comparison, the material and cavity parameter are kept constant at $\gamma = 2.5 \times 10^9 s^{-1}$ and $\Gamma_c = 10^{11} s^{-1}$, while the pump values are adapted to explore the transition region between spontaneous and stimulated emission: $P = 2.05$ ($\beta = 10^{-4}$); $P = 0.22$ ($\beta = 10^{-3}$); $P = 0.025$ ($\beta = 10^{-2}$); $P = 2 \times 10^{-3}$ ($\beta = 10^{-1}$). The respective photon numbers are rescaled by the factor indicated next to each trace (for better comparison) and the traces are shifted vertically for clarity.

As mentioned in the main text, spikes are predicted in the laser output by the SS [13]. Their presence for the mesoscale laser we are investigating ($\beta = 10^{-4}$) can be

interpreted on the basis of the absence of photons (at low pump values) in certain time intervals, due to the repartition of the spontaneous events on the ensemble of e.m. cavity modes. Since stimulated emission is initiated by a spontaneous photon present in the lasing mode, in its absence no lasing can take place, and thus, it is reasonable to expect to find, at times, zero photons in the output.

The presence of a laser spike can also be understood in terms of strong transient gain – due to the accumulation of dipole population in the absence of its conversion into stimulated emission –, which produces an overuse of the stored energy, thus a drop into the coherent photon number until extinction of the laser field. Thus, in this regime it is reasonable to expect laser bursts, which are not consistent with the picture, developed for Class A lasers (e.g., [15]), where a "stimulated photon fraction" increases steadily in the threshold region. The concept of a stationary regime is meaningful in the Class A laser since the laser field evolution takes place only along one dynamical dimension, while the intrinsic 2-D nature of the phase space (cf. Section X) allows for dynamically more complex behaviour.

The next question is whether such a regime is specific to mesoscale devices or whether it can carry over to nanolasers. An answer can be obtained by comparing the results obtained from the SS for β values consistent with a nanolaser. The proof that we are providing here is purposefully chosen to maintain physical conditions which are as close as possible to those of the mesoscale laser, in order to find a possible scaling. Since the SS is used here as a proof of principle, without including the details of each specific meso- or nanolaser, maintaining common parameter values is indispensable for the internal self-consistency of the prediction.

Fig. 5 shows the output dynamics of the laser computed after transmission from the output mirror for different values of β (all other parameters are kept constant, only the pump value is adjusted to compute the dynamics in the threshold transition region – cf. Fig. 2 of [13]). The predictions of the Stochastic Simulator show that a spiking regime exists throughout the mesoscale and well into the nanoscale range of laser sizes. The change in amplitude (cf. caption for comment) is trivially related to the smaller photon number, while the increase in the spiking frequency is understandable in terms of the growing fraction of spontaneous emission appearing in the laser mode, which render the conversion of spontaneous into stimulated emission more likely as the number of e.m. cavity modes is reduced.

While nanolasers with even larger values of β may not display the same kind of spiking, we see nonetheless that this regime persists even when the number of cavity modes is strongly reduced. Thus, it is not related to the presence of a large number of e.m. cavity modes (e.g., $N > 100$), but to their mere presence. In addition to its fundamental significance, the existence of spiking even in nanolasers may pave the way for new kinds of applications.

[1] Thorlabs catalogue page: http://www.thorlabs.de/catalogpages/V21/1439.PDF
Manual: http://www.thorlabs.de/thorcat/15900/LDC200CV-Manual.pdf

[2] Thorlabs catalogue page: http://www.thorlabs.de/thorcat/8700/PDA8GS-SpecSheet.pdf

[3] G. Shtengel, H. Temkin, T. Uchida, M. Kim, P. Brusenbach, and C. Parsons, Spontaneous emission factor and its scaling in vertical cavity surface emitting lasers, *Appl. Phys. Lett.* **64**, 1062 (1994).

[4] Thorlabs catalogue page: http://www.thorlabs.de/ catalogpages/V21/1243.PDF
Manufacturer's specifications: http://www.thorlabs.de/thorcat/11900/VCSEL-980-MFGSpec.pdf

[5] J.H. Shin, Y.G. Ju, H.E. Shin, and Y.H. Lee, Spontaneous emission factor of oxidized vertical-cavity surface-emitting lasers from the measured below-threshold cavity loss, *Appl. Phys. Lett.* **70**, 2344 (1997).

[6] K.J. Vahala, Optical microcavities, *Nature* **424**, 839 (2003).

[7] R. Michalzik and K.J. Ebeling, Operating principles of VCSELs. In: Vertical-Cavity Surface-Emitting Laser Devices, pp. 53-98. H.E. Li and K. Iga Eds. (Springer, Berlin & Heidelberg,2003).

[8] A.E. Siegman, Lasers, (University Science Books, Mills Valley, CA 1986).

[9] L.A. Coldren and S.W. Corzine, *Diode Lasers and Photonic Integrated Circuits*, (Wiley, New York, 1995).

[10] S. Adachi, GaAs, AlAs, and Al_x Ga_{1x} As: Material parameters for use in research and device applications, *J. Appl. Phys.* **58**, R1 (1985).

[11] G.L. Lippi, S. Barland, N. Dokhane, F. Monsieur, P.A. Porta, H. Grassi, and L.M. Hoffer, Phase space techniques for steering laser transients, *J. Opt. B: Quantum Semiclass. Opt.* **2**, 375 (2000).

[12] X. Hachair, S. Barland, J.R. Tredicce, and G.L. Lippi, Optimization of the switch-on and switch-off transition in a commercial laser, *Appl. Opt.* **44** (22), 4761 (2005).

[13] G.P. Puccioni and G.L. Lippi, Stochastic Simulator for modeling the transition to lasing, *Opt. Express* **23** (3), 2369 (2015).

[14] J.R. Tredicce, F.T. Arecchi, G.L. Lippi, and G.P. Puccioni, Instabilities in lasers with an injected signal, *J. Opt. Soc. Am.* **B 2**, 173 (1985).

[15] F.T. Arecchi and V. Degiorgio, Statistical Properties of Laser Radiation During a Transient Buildup, *Phys. Rev. A* **3**, 1108 (1971).
